# What an rRNA Secondary Structure Tells about Phylogeny of Fungi in Ascomycota with Emphasis on Evolution of Major Types of Ascus

**DOI:** 10.1371/journal.pone.0047546

**Published:** 2012-10-26

**Authors:** Wen-Ying Zhuang, Chao-Yang Liu

**Affiliations:** 1 State Key Laboratory of Mycology, Institute of Microbiology, Chinese Academy of Sciences, Beijing, People's Republic of China; 2 School of Life Science and Engineering, Southwest University of Science and Technology, Mianyang, People's Republic of China; Nanjing Agricultural University, China

## Abstract

**Background:**

RNA secondary structure is highly conserved throughout evolution. The higher order structure is fundamental in establishing important structure-function relationships. Nucleotide sequences from ribosomal RNA (rRNA) genes have made a great contribution to our understanding of Ascomycota phylogeny. However, filling the gaps between molecular phylogeny and morphological assumptions based on ascus dehiscence modes and type of fruitbodies at the higher level classification of the phylum remains an unfulfilled task faced by mycologists.

**Methodology/Principal Findings:**

We selected some major groups of Ascomycota to view their phylogenetic relationships based on analyses of rRNA secondary structure. Using rRNA secondary structural information, here, we converted nucleotide sequences into the structure ones over a 20-symbol code. Our structural analyses together with ancestral character state reconstruction produced reasonable phylogenetic position for the class Geoglossomycetes as opposed to the classic nucleotide analyses. Judging from the secondary structure analyses with consideration of mode of ascus dehiscence and the ability of forming fruitbodies, we draw a clear picture of a possible evolutionary route for fungal asci and some major groups of fungi in Ascomycota. The secondary structure trees show a more reasonable phylogenetic position for the class Geoglossomycetes.

**Conclusions:**

Our results illustrate that asci lacking of any dehiscence mechanism represent the most primitive type. Passing through the operculate and *Orbilia*-type asci, bitunicate asci occurred. The evolution came to the most advanced inoperculate type. The ascus-producing fungi might be derived from groups lacking of the capacity to form fruitbodies, and then evolved multiple times. The apothecial type of fruitbodies represents the ancestral state, and the ostiolar type is advanced. The class Geoglossomycetes is closely related to Leotiomycetes and Sordariomycetes having a similar ascus type other than it was originally placed based on nucleotide sequence analyses.

## Introduction

As widely used markers in phylogenetic study, the ribosomal RNA (rRNA) genes have made a huge contribution to exploration of deep relationships among fungi. In recent years, there has been continuous recognition that higher order structure is fundamental in establishing structure-function relationships in biological macromolecules [1]–[4]. Presently, secondary structure information is mainly used to build a reliable sequence alignment [5]–[6] or to offer a more realistic model of evolution [7]–[9]. Therefore, secondary structure has only been used indirectly because the phylogeny recovered is based solely on nucleotide comparison.

It is well known that a complex rRNA secondary structure consists of base paired stem and unpaired loop regions. The pairing within stems involves both Watson-Crick (A∶U, G∶C) and noncanonical pairs. Due to the constraints arising from having to conserve structure, evolution of sequences in stems may differ from those in the loop regions. Therefore, the diversity in nucleotide pairings and evolutionary divergence between stem and loop should be considered when using rRNA sequences for phylogeny estimation.

To address these issues, a new coding method based on the positions and the types of base pairs has recently been developed [10]–[11]. By using more symbol codes and more complex substitution matrices, this approach has demonstrated superiority over the traditional nucleotide analysis.

Ascus production is a very important criterion in fungal taxonomy, and is used to delimit the largest and the most complex phylum, the Ascomycota, in the Kingdom Fungi [12]. Fungi of this phylum show the highest species diversity and morphological variation, have a wide range of ecological niches, live as saprophytes, parasites and symbionts, and influence the daily life of human beings.

Ascus dehiscence mechanisms have been used as important taxonomic criterion for high level classification of Ascomycota [13]–[18]. Several ascus dehiscence modes were examined through an understanding of their ultrastructure details [19]–[25]. Type of fruitbody as a phenotypic feature is also frequently used in taxonomic systems [16]–[18], [26]. The phylogenies of fungi in Ascomycota have been reconstructed based on single- or multi-gene analyses, and as a result several higher level taxa were recently established, such as Geoglossomycetes, Neolectomycetes, Orbiliomycetes, etc. [27]–[32].

However, for all cases described above, phylogenetic analyses in Ascomycota were still based on the 4-bases variation (A, C, G, T against A, C, G, T) across sites, while more complex base pair changes were seldom considered. We thus treated, in this study, the rRNA secondary structure as a potentially phylogenetic signal, and converted the nucleotide sequence into structure-coding sequence. In order to evaluate the effects of structure information on tracing phylogeny among some groups of Ascomycota and the evolution of major types of asci, we made an objective comparison of phylogeny generated by structure analyses against nucleotide approaches.

## Results

### Phylogenetic analyses

Among the ascus-producing fungi investigated, taxa of 12 classes, Taphrinomycetes, Schizosaccharomycetes, Neolectomycetes, Saccharomycetes, Pezizomycetes, Orbiliomycetes, Dothideomycetes, Lecanoromycetes, Eurotiomycetes, Geoglossomycetes, Leotiomycetes and Sordariomycetes, were examined based on the nucSSU+nucLSU dataset. The structure sequences were produced by using a coding method which is similar to that used by Smith et al. [10]. Four symbols were used for unpaired bases and 16 for the paired ones.

**Figure 1:**
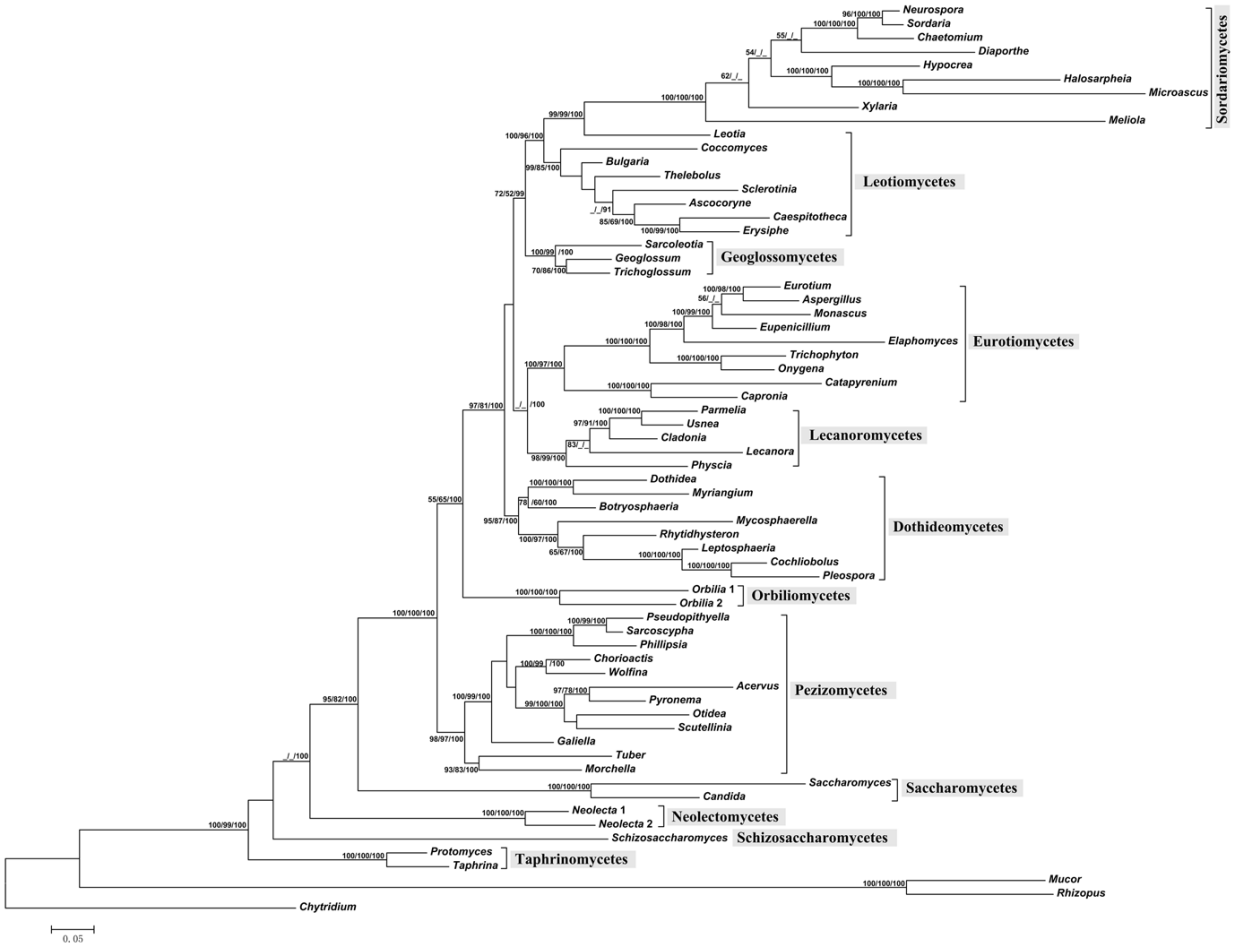
Phylogeny of 12 Ascomycota classes inferred from maximum likelihood analysis based on rRNA secondary structure signature. From left to right, numbers at nodes showing MLBP (>50%), MPBP (>50%) and BIPP (>90%), respectively.

**Figure 2:**
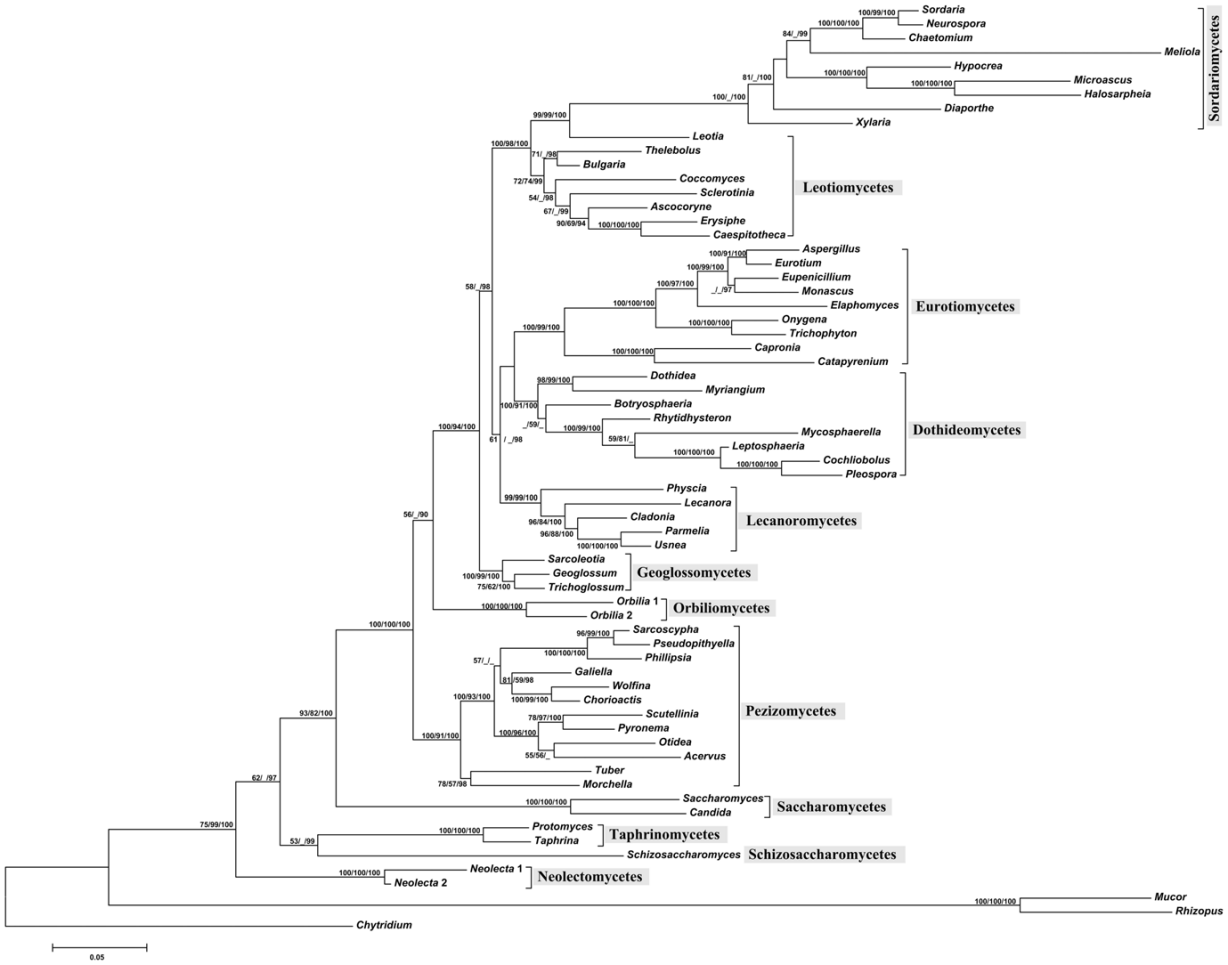
Phylogeny of 12 Ascomycota classes inferred from nucleotide sequences using maximum likelihood approach. From left to right, numbers at nodes showing MLBP (>50%), MPBP (>50%) and BIPP (>90%), respectively.

In general, the secondary structure tree (Figure 1) shared a very similar topology, as did the topology based on the nucleotide sequences (Figure 2) in an ML framework. The resulting MLBP (>50%), MPBP (>50%), and BIPP (>90%) are shown at internal branches from left to right, respectively.

Based on the nucSSU+nucLSU phylogeny, Ascomycota was shown to be a monophyletic group, and received very high statistical supports for the secondary structure sequence analyses (100% MLBP, 99% MPBP, and 100% BIPP); and a relatively high supports for the correlated nucleotide analyses (75% MLBP, 99% MPBP, and 100% BIPP).

**Figure 3:**
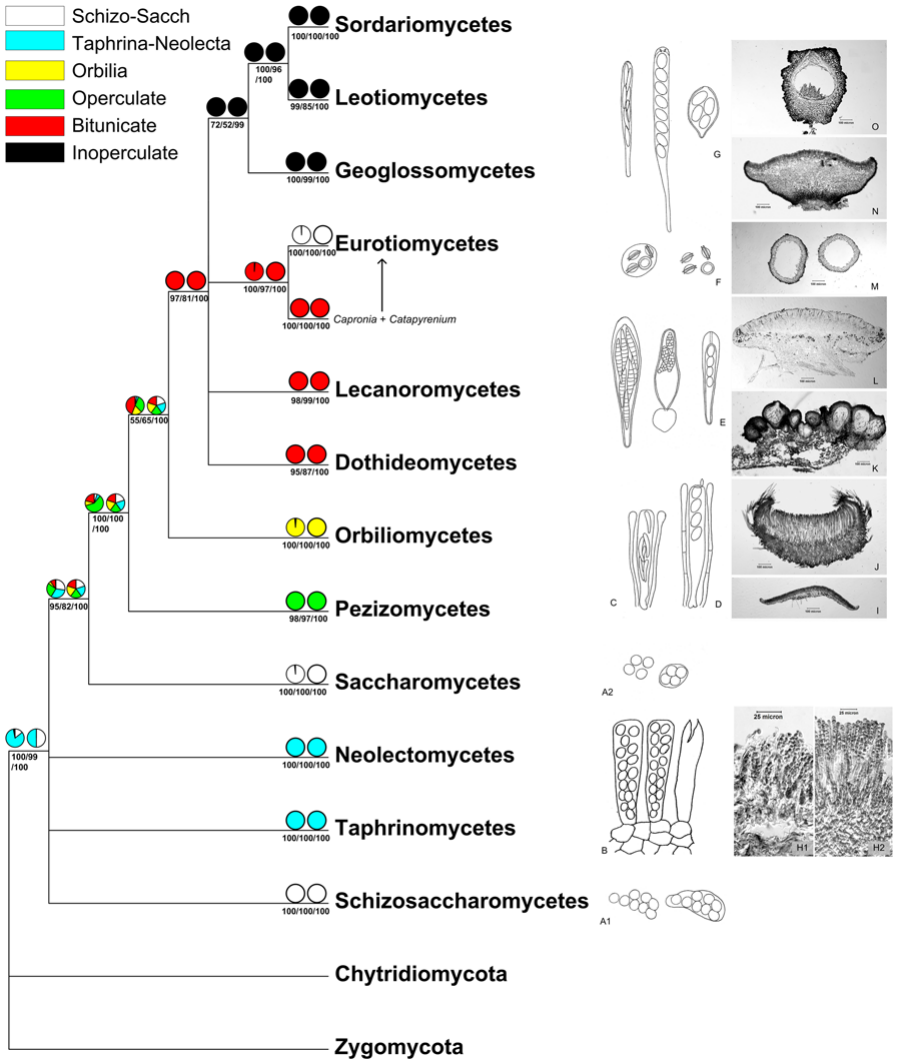
Ancestral state reconstruction of ascus dehiscence mechanism based on rRNA secondary structure signatures. Character states were reconstructed using maximum likelihood and maximum parsimony methodsbased on a best ML structure tree. From left to right, the pie charts above internal branches resulted from the ML and MP analyses; the corresponding probabilities (MLBP, MPBP, BIPP) for the nodes were shown below internal branches. A. *Schizosaccharomyces*-*Saccharomyces* type of asci, A1 from *Schizosaccharomyces octosporus* (Alexopoulos et al., 1996), A2 from *Saccharomyces* sp. B, H. *Taphrina*-*Neolecta* type of asci; H1. naked asci of *Taphrina pruni* seated on host tissue, H2. exposed asci of *Neolecta irregularis* arising from subhymenial tissue. C, I. *Orbilia*-type of ascus, and apothecium of *Orbilia auricolor*. D, J. Operculate ascus, and apothecium of *Trichophaea* sp. E, K, L. Bitunicate asci, from left to right: *Saccardoella* sp., *Myriangium bambusae* (Tai, 1931), *Lecanora* sp., and pseudothecia of B*yssosphaeria alnea* and apothecium of *Lecanora* sp. F, M. *Schizosaccharomyces*-*Saccharomyces* type of ascus, and cleistothecia of *Eupenicillium* sp. G, N, O. Inoperculate asci, from left to right: *Lanzia huangshanica*, *Sordaria* sp., *Erysiphe* sp., and apothecium of *Calycellinopsis Xishuangbanna* and perithecium of *Bertia macrospora* var. *tetraspora*.

The ancestral groups, including Schizosaccharomycetes, Saccharomycetes, Taphrinomycetes and Neolectomycetes, represented by species of the genera *Schizosaccharomyces*, *Saccharomyces*, *Candida*, *Protomyces*, *Taphrina* and *Neolecta*, were located at the base of the phylogenetic trees (Figures 1, 2). They are not able to produce fruitbodies (Figure 3A, B, H, P), except for fungi of Neolectomycetes. In Neolectomycetes a simple clavate ascoma gives rise to asci in a palisade layer that lacks any accompanying sterile elements. The remaining taxa of Ascomycota were resolved again as monophyletic clade with full supports in both approaches (MLBP = 100%, MPBP = 100%, and BIPP = 100%).

In all analyses, Pezizomycetes releasing spores by an apical lid formed a monophyletic group with high supports (BP >95%, BIPP = 100%), and was related to but more advanced than the ancestral classes (Figures 1, 2).

In both cases (structure and nucleotide trees), Orbiliomycetes with ascus dehiscence results from a tearing of the flattened apex of the ascal wall [19] represented an independent monophyletic lineage (100% BP and PP), whereas its phylogenetic relationship with the rest groups of Ascomycota is uncertain due to the insufficient supports (55% MLBP, 65% MPBP, and 100% BIPP for structure analyses and 56% MLBP, <50% MPBP, and 90% BIPP for nucleotide analyses). Fungi of the remaining classes produce bitunicate and inoperculate asci. They appeared to be even more developed or advanced groups (Figures 1, 2).

As to the relationships among Dothideomycetes and Lecanoromycetes, both producing bitunicate asci, and Eurotiomycetes with asci deliquescent to release ascospores were not clearly resolved or received poor to no support (<50% MLBP, <50% MPBP) although three classes were strongly supported as monophyletic (MLBP >90%, MPBP >85% and BIPP = 100%). In any cases, fungi in Leotiomycetes and Sordariomycetes are closely related (MLBP = 100%, MPBP >95%, and BIPP = 100%), and share a similar type of ascus dehiscence mechanism.

Topological incongruence was found between the rRNA secondary structure phylogeny and the traditional one based on nucleotides. For example, the genera *Geoglossum* and *Trichoglossum*, the earth tongue fungi, – as the representative of Geoglossomycetes and having inoperculate asci – are always shown as an independent lineage. In the secondary structure tree, Geoglossomycetes turned out to be associated with Leotiomycetes and Sordariomycetes possessing the same type of asci (72% MLBP, 52% MPBP and 100% BIPP) (Figure 1). In contrast, in the nucleotide trees it was located between Orbiliomycetes and the remaining Ascomycota, and showed close relationship to the latter groups (100% MLBP, 94%MPBP and 100% BIPP) (Figure 2).

### Analyses of character evolution

**Figure 4:**
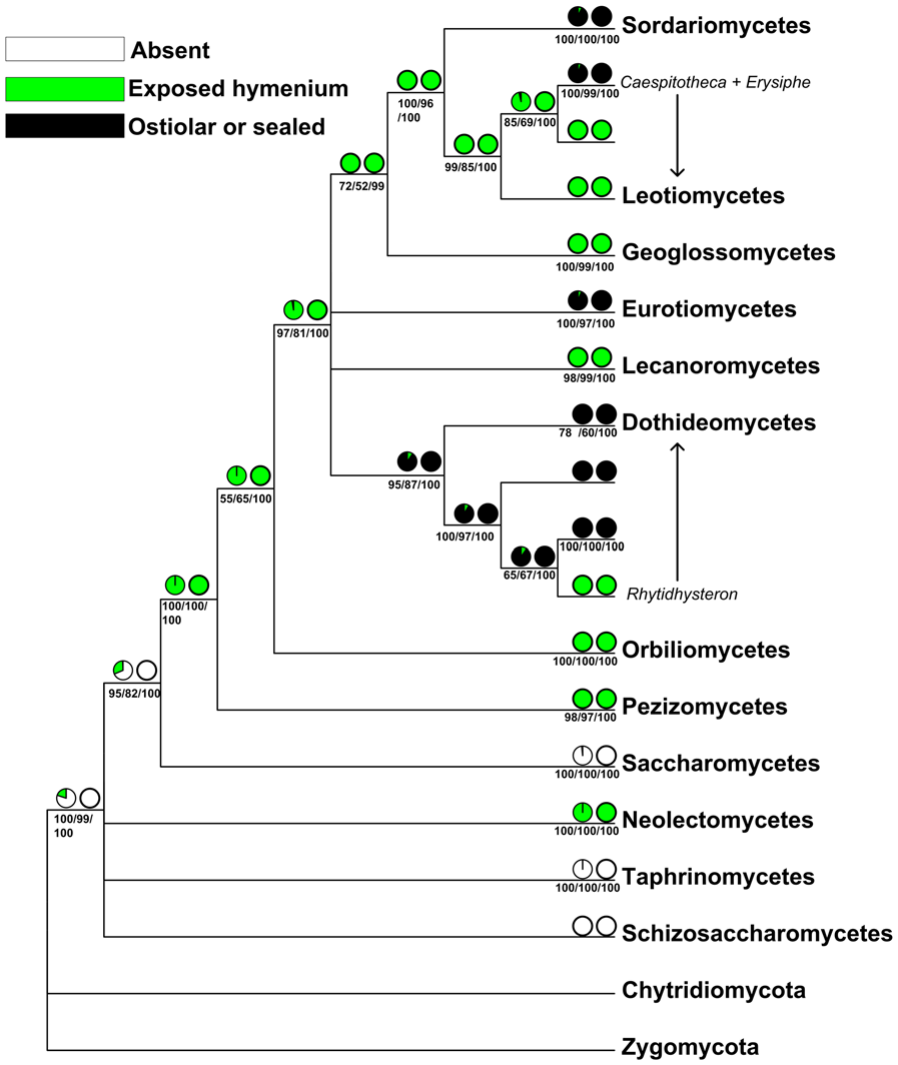
Ancestral state reconstruction of ascoma shape for 12 classes of Ascomycota based on rRNA secondary structure signatures. Character states were reconstructed using maximum likelihood and maximum parsimony methods based on a best ML structure tree. From left to right, the pie charts above internal branches resulted from the ML and MP analyses; the corresponding probabilities (MLBP, MPBP, BIPP) for the nodes were shown below internal branches.

The results of the ancestral state reconstruction of ascus dehiscence mechanism and ascoma shape are shown in Figures 3 and 4.

The ASR of ascus dehiscence mechanism revealed roughly a single origin of the six character states (Figure 3). The *Schizosaccharomyces*-*Saccharomyces* type and *Taphrina*-*Neolecta* type were recovered as the ancestral stage during the evolution of fungal ascus. The *S*c*hizosaccharomyces*-*Saccharomyces* ascus type evolved once, which occurred again in Eurotiomycetes possessing a sealed fruitbody. The operculate and *Orbilia*-type asci developed successively at a later stage. After branching of Orbiliomycetes, bitunicate asci became the ancestral state for the remaining taxa. The inoperculate type of ascus typically shared by Leotiomycetes, Sordariomycetes and Geoglossomycetes appeared to be the most advance type.

The three character states of the character ascoma shape revealed a seemingly monophyletic pattern of evolution (Figure 4). Among the fungi investigated, taxa lacking of ascomata were reconstructed as ancestral groups. Those with exposed hymenium came up later, which were found in five phylogenetic lineages, i.e. Neolectomycetes, Orbiliomycetes, Pezizomycetes, Lecanoromycetes and Leotiomycetes (except for *Caespitotheca* and *Erysiphe*). Fungi sharing ostiolar or sealed fruitbodies represented the most advance form, which include Dothideomycetes, Eurotiomycetes and Sordariomycetes. This trait evolved independently at least three times.

## Discussion

### Phylogeny of Ascomycota inferred form rRNA secondary structure signals

As mentioned earlier, phylogeny of 12 classes of Ascomycota based on secondary structure sequences was found to be generally similar to that inferred from the correlated nucleotide sequences. For example, Taphrinomycetes, Schizosaccharomycetes, Saccharomycetes and Neolectomycetes are the basal groups, and Leotiomycetes and Sordariomycetes represent the advanced groups (Figures 1, 2). Minor divergences in the phylogenetic trees were shown in between the basal and advanced groups. Combining our secondary structure results with the ascus dehiscence mechanism and shape of ascoma suggests a reasonable change in the phylogenic relationships, compared to that based on the nucleotide analyses. The placements of the class Geoglossomycetes is a good example of the changes.

The earth tongue fungi represented by the genera *Geoglossum* and *Trichoglossum* are characterized by simple, clavate and stipitate fruitbodies lacking ectal excipulum, inoperculate asci which are surrounded by well-developed paraphyses, and elongate, brown and septate ascospores. Due to having a similar type of ascus dehiscence mechanism, they were traditionally placed as members of the Leotiomycetes [33].

Recently, separation of the earth tongue fungi from other inoperculate taxa has been observed with establishment of the class Geoglossomycetes [34]. This class is shown to be related either to Eurotiomycetes and Lecanoromycetes [30] or to Orbiliomycetes and Dothidiomycetes [35]. A similar situation is also found in our (traditional) independent-sites trees (Figure 2). However, the structure sequence analyses using ML, MP and BI methods clearly draw a different picture, in which the class Geoglossomycetes is affiliated with fungal classes having a similar type of asci and ascus dehiscence mechanism, the Leotiomycetes and Sordariomycetes (Figures 1, 3). This result is congruent with the six-locus phylogeny inferred from the combined 18S, 28S and 5.8S rDNA, EF-1α, RPB1 and RPB2 sequence analysis [36]. Based on the evolution and function of ascus, secondary structure phylogeny may provide a more realistic position of this class. Likewise, as revealed by morphological features such as spore discharge through an apical pore, i.e. the ascus apical apparatus and its ultrastructure, as well as the simple and clavate fruitbodies lacking of ectal excipular tissue appeared in some genera, the class Geoglossomycetes seemingly serves as the basal group of the fungi producing inoperculate asci (Figures 1, 3). Presence of the simple clavate fruitbody in fungi of Geoglossomycetes recalls that of Neolectomycetes, one of the most ancestral groups. However, the former class is obviously advanced due to its capacity of producing abundant sterile paraphyses surrounding asci, which is never found in Neolectomycetes. Considering the fact that Neolectomycetes represents the ancestral state of fungi producing fruitbody, Geoglossomycetes is thus logically treated as basal group of fungi forming inoperculate asci.

### Ancestral character state reconstruction

#### Ascus dehiscence mechanism and evolution

As the reproductive organ of fungi in Ascomycota, asci are functionally critical. They are where karyogamy and meiosis take place, ascospores are produced, and genetic information is passed on. The development, wall structure, and dehiscence mechanism of asci have long been used as a taxonomic criterion at higher ranks, and play an essential rule in fungal systematics [13]–[16], [33], [37]–[39].

Attempts have been made in the past decades to investigate the relationship between behavior and structure of ascus and taxonomy [19]–[25], [33], [40]–[43]. However, little in-depth research was performed on the evolution of fungal asci, although previous work has provided valuable information [30], [44]. In this study, we utilize ancestral character state reconstruction to trace ascus evolution based on rRNA secondary structure. The mapping of the six ascus dehiscence characters on the maximum likelihood tree draws an outline of possible evolutionary route of fungal asci (Figure 3).

The genera *Protomyces*, *Taphrina*, *Schizosaccharomyces*, *Saccharomyces*, *Candida* and *Neolecta* are representatives of four classes, and are the basal groups in Ascomycota (Figure 3). These fungi include morphologically and ecologically diverse groups. *Schizosaccharomyces* (Schizosaccharomycetes) and *Saccharomyces* (Saccharomycetes) are characterized by vegetative fission or vegetative budding, respectively. *Taphrina* and *Protomyces* are plant-parasites and not able to produce any fruitbody, while *Neolecta* is mainly saprophytic and with asci in a palisade layer seating on a simple clavate fruitbody.

Obviously, fungal asci lacking of characterized dehiscence mechanism appear to be primitive. Our results showed support for a *S*c*hizosaccharomyces*-*Saccharomyces* or *Taphrina*-*Neolecta* character state as ancestral to the majority of superclass nodes (100% MLBP, 99% MPBP and 100% BIPP) (Figure 3). *S*c*hizosaccharomyces*-*Saccharomyces* ascus type is characterized by naked asci without any surrounding hyphae or peridium and by deliquescent release of ascospores (Figure 3A1, A2). This ancestral character state may represent independent origin, and evolve once after the branching of Saccharomycetes, as it occurred again in some members of Eurotiomycetes.

In contrast to the *S*c*hizosaccharomyces*-*Saccharomyces* type of asci, the *Taphrina*-*Neolecta* type is elongate, has a distinguishable apical and basal portion,and releases spores by an irregular apical slit [23], [25] (Figure 3B, H). However, it seems to be difficult to draw a conclusion that this ascus type is of independent origin, because the relationship between Taphrinomycetes and Neolectomycetes was not well resolved due to the low statistical support (Figure 1).

The rest of character states involved are operculate type, *Orbilia-*type, bitunicate type and inoperculate type of asci. Generally, each character state is correlated with specific classes (Figure 3). The ascus types of all remaining classes of Pezizomycotina (Orbiliomycetes, Pezizomycetes, Dothideomycetes, Lecanoromycetes, Eurotiomycetes, Geoglossomycetes, Leotiomycetes and Sordariomycetes) forming a highly supported monophyletic group (100% MLBP, MPBP and BIPP) are capable of forcible spore discharge except for Eurotiomycetes.

Fungi of Pezizomycetes have long been recognized by their operculate asci which discharge spores through an apical lid, and are associated with sterile elements [13], [14], [33] (Figure 3D). They form a highly supported class. Morphologically, the ability of forcible spore discharge through an apical lid is developed in comparison with that by irregular slit in the very primitive groups.

The genus *Orbilia* (Orbiliomycetes) is characterized by their small and semitranslucent, apothecial fruitbodies (Figure 3I), very short asci arising from croziers, with a truncate apex and accompanied by paraphyses (Figure 3C). This group has long been regarded as an independent family (Orbiliaceae) of Helotiales, because its asci were thought to be inoperculate [16], [33], [45]. On conducting an ultrastructure study via electron microscopy, it was discovered that a pore does not exist at the truncate ascus apex. Instead, ascus dehiscence results from a tearing of the flattened apex of the ascal wall [19].

It indicates that operculate and *Orbilia* asci are evolved compared with those produced by *Taphrina*-*Neolecta*, as their asci arise from crosiers and are associated with numerous paraphyses. Additionally these asci are formed within a well-organized apothecial fruitbody with ectal excipulum (Figure 3I, J), in contrast to the naked and completely exposed *Taphrina*-*Neolecta* asci with absence of any paraphyses.

After the branching of Orbiliomycetes, bitunicate type of asci was supported as an ancestral state for rest of the taxa with BIPP = 100% and BP ranging from 81% to 97%. This ascus type seems to be of a monophyletic origin. Bitunicate asci are characterized by a clearly differentiated rigid outer wall and elastic inner wall. The ascus inner wall protrudes beyond the outer wall during spore discharge (Figure 3E). A pore-like rupturing appears at the apical portion of the extending inner wall to set the spores free. Dothideomycetes and Lecanoromycetes are representatives of this ascus type. Previous RPB2 Bayesian phylogeny indicates that asci produced by Lecanoromycetes are closely related to the unitunicate ones and distantly related to the bitunicate asci [44]. This opinion is only partially supported by our results (Figure 3).

Inoperculate type of ascus is usually able to forcibly discharge spores through an apical pore (Figure 3G). Pore structure varies between groups and sometimes even among species of the same genus [21], [33], [43]. Our results indicate that inoperculate type is derived from bitunicate type.

Our results partially agree with the previous hypothesis of ascus evolution based on the RPB2 phylogeny; that prototunicate and unitunicate (including operculate and inoperculate) asci evolved early and the bitunicate type is advanced [32], [44]. Based on the reconstruction of ascus ancestral character states, indeed, operculate asci are primitive compared to the bitunicate ones, because Pezizomycetes and Orbiliomycetes form an early-diverging lineage at the base.

In the (traditional) independent-sites trees as well as that shown in some previous studies, Geoglossomycetes is divergent from other fungi possessing inoperculate asci, and associated with those bearing bitunicate asci [30], [35] (Figure 2). In contrast, our current results of the ancestral state reconstruction based on structure information do not support the above hypothesis that inoperculate character state is as ancestral state for the bitunicate one, since the Geoglossomycetes is affiliated with fungi having a similar type of asci and stands as an independent lineage (Figure 1). As revealed by morphological features such as the ascus apical apparatus and its ultrastructure, it is reasonable that *Geoglossum* and *Trichoglossum* having a simple clavate fruitbody may serve as the basal group of the fungi producing inoperculate asci.

On rare occasions a connection between operculate asci and the bitunicate type was found. We observed in some samples of *Pseudopithyella minuscula* (a member of Pezizomycetes typically releasing spores by an apical lid) that its inner wall of a few asci protruding beyond the outer wall, which behaves just like bitunicate asci [46]. This might suggest an evolutionary connection between the operculate and bitunicate types. It recurs in our results based on the secondary structure information (Figure 3).

To summarize, our results provide a view of the possible evolutionary trend of some major types of fungal asci. *Schizosaccharomyces-Saccharomyces* type and the *Taphrina*-*Neolecta* one are seen as being the most ancestral. These types followed by the operculate type and *Orbilia*-type, then the bitunicate type, and finally, the most evolved inoperculate type.

#### Ascocarp evolution

Ascoma shape has long been used in classification of Ascomycota [12], [47]. This trait seems to be good in tracing the origin and evolution of fruitbody types. The combination of morphology/anatomy, structure sequence analyses, and ancestral character state reconstruction provides a possible development of fruitbody in Ascomycota (Figure 4).

It is clear that the ascus-producing fungi were originated from the groups (Schizosaccharomycetes, Saccharomycetes, Taphrinomycetes) lacking of capacity to form fruitbodies (Figure 4), that represent the very primitive stage of development. Through formation of a rudimentary layer of hymenium (Taphrinomycetes) directly on the substrates (Figure 3H1), a simple clavate fruitbody with completely open hymenium (Neolectomycetes) evolved (Figure 3H2).

Compared to the previous hypothesis that apothecial ascomata are the primitive type of fruitbody, and perithecia and cleistothecia are derived forms [16], our results support the hypothesis, and demonstrate a shift from the apothecial ancestors (characterized by exposed hymenium) to the ostiolar or sealed ascomata (Figure 4). Nevertheless, it should be noted that all taxa possessing apothecial fruitbodies do not group together to form an early divergent lineage, although a monophyletic origin of apothecial fruitbodies is found in this study. In contrast, the apothecial type distributes across Neolectomycetes, Orbiliomycetes, Pezizomycetes, partial of Dothideomycetes, Lecanoromycetes, Geoglossomycetes and Leotiomycetes (Figures 3I, J, L, N, 4).

Although the ostiolar or sealed ascomata derived from the apothecial ancestors, independent origins of this trait have been assumed by our data.

To draw our conclusion, the apothecial type of fruitbodies originated first and represents the primitive form, and the ostiolar ones are advanced.

## Materials and Methods

### Taxon sampling

66 nuclear small and large subunit ribosomal DNA (nucSSU and nucLSU rDNA) sequences representing 12 classes of Ascomycota were obtained from GenBank. A species of Chytridiomycota and two of Zygomycota were selected as outgroup taxa (Table 1).

**Table 1:** Taxa investigated in this study.

Higher level ranks	SSU		LSU	
Ascomycota	Species	GenBank acc. no.	Species	GenBank acc. no.
**Sordariomycetes**	*Chaetomium globosum*	AY545725	*Chaetomium globosum*	AAFU01000611
	*Diaporthe* sp.	AB245446	*Diaporthe angelicae*	AY196781
	*Halosarpheia retorquens*	AF050486	*Halosarpheia japonica*	HQ009884
	*Hypocrea rufa*	AY489694	*Hypocrea jecorina*	AF510497
	*Meliola niessleana*	AF021794	*Meliola variaseta*	EF094840
	*Microascus cirrosus*	M89994	*Microascus trigonosporus*	DQ470958
	*Neurospora crassa*	X04971	*Neurospora crassa*	FJ360521
	*Sordaria fimicola*	X69851	*Sordaria fimicola*	AY545728
	*Xylaria carpophila*	Z49785	*Xylaria hypoxylon*	AY544648
**Leotiomycetes**	*Ascocoryne solitaria*	DQ002904	*Ascocoryne sarcoides*	FJ176886
	*Bulgaria inquinans*	AJ224362	*Bulgaria inquinans*	DQ470960
	*Caespitotheca forestalis*	AB193465	*Caespitotheca forestalis*	AB193467
	*Coccomyces dentatus*	AY544701	*Coccomyces strobi*	DQ470975
	*Erysiphe mori*	AB033484	*Erysiphe pisi*	CACM01000006
	*Leotia lubrica*	AY544687	*Leotia lubrica*	AY544644
	*Sclerotinia sclerotiorum*	AY187065	*Sclerotinia sclerotiorum*	DQ470965
	*Thelebolus ellipsoideus*	DQ067574	*Thelebolus ellipsoideus*	FJ176895
**Geoglossomycetes**	*Geoglossum nigritum*	AF113716	*Geoglossum nigritum*	AY544650
	*Sarcoleotia globosa*	AY789298	*Sarcoleotia globosa*	AY789428
	*Trichoglossum hirsutum*	AY544697	*Trichoglossum hirsutum*	AY544653
**Eurotiomycetes**	*Aspergillus penicillioides*	AB002060	*Aspergillus protuberus*	FJ176897
	*Capronia coronata*	AJ232939	*Capronia mansonii*	AY004338
	*Catapyrenium lachneum*	AF412410	*Catapyrenium cinereum*	EF643747
	*Elaphomyces maculatus*	U45440	*Elaphomyces guangdongensis*	HM357248
	*Eupenicillium crustaceum*	D88324	*Eupenicillium ochrosalmoneum*	EF626957
	*Eurotium rubrum*	U00970	*Eurotium* sp.	FR848827
	*Monascus purpureus*	DQ782881	*Monascus purpureus*	DQ782908
	*Onygena equina*	U45442	*Onygena corvina*	FJ358287
	*Trichophyton rubrum*	X58570	*Trichophyton equinum*	ABWI01001612
**Lecanoromycetes**	*Cladonia rangiferina*	AF184753	*Cladonia stipitata*	DQ973026
	*Lecanora dispersa*	L37734	*Lecanora contractula*	DQ986746
	*Parmelia saxatilis*	AF117985	*Parmelia saxatilis*	AY300849
	*Physcia aipolia*	AF241542	*Physcia aipolia*	DQ782904
	*Usnea florida*	AF117988	*Usnea strigosa*	DQ973033
**Dothideomycetes**	*Botryosphaeria ribis*	U42477	*Botryosphaeria stevensii*	DQ678064
	*Cochliobolus sativus*	U42479	*Cochliobolus sativus*	DQ678045
	*Dothidea sambuci*	AY544722	*Dothidea insculpta*	DQ247802
	*Leptosphaeria maculans*	DQ470993	*Leptosphaeria maculans*	DQ470946
	*Mycosphaerella mycopappi*	U43463	*Mycosphaerella pneumatophorae*	FJ176856
	*Myriangium duriaei*	AY016347	*Myriangium duriaei*	DQ678059
	*Pleospora herbarum*	U05201	*Pleospora* sp.	EF177848
	*Rhytidhysteron rufulum*	AF201452	*Rhytidhysteron rufulum*	GU397353
**Pezizomycetes**	*Acervus epispartius*	DQ787814	*Acervus epispartius*	DQ220305
	*Chorioactis geaster*	AF104340	*Chorioactis geaster*	AY307945
	*Galiella rufa*	AF004948	*Galiella rufa*	FJ176869
	*Morchella esculenta*	U42642	*Morchella* cf. *elata*	AY544665
	*Otidea leporina*	DQ248955	*Otidea leporina*	DQ220386
	*Phillipsia domingensis*	AF006315	*Phillipsia olivacea*	AY945843
	*Pseudopithyella minuscula*	AF006317	*Pseudopithyella minuscula*	AY544658
	*Pyronema domesticum*	U53385	*Pyronema domesticum*	DQ247805
	*Sarcoscypha coccinea*	AY544691	*Sarcoscypha coccinea*	FJ176859
	*Scutellinia korfiana*	DQ787829	*Scutellinia scutellata*	DQ247806
	*Tuber gibbosum*	U42663	*Tuber gibbosum*	FJ176877
	*Wolfina aurantiopsis*	AF104664	*Wolfina aurantiopsis*	AY945859
**Orbiliomycetes**	*Orbilia auricolor*	DQ471001	*Orbilia auricolor*	DQ470953
	*Orbilia delicatula*	U72603 1	*Orbilia delicatula*	AY261178
**Neolectomycetes**	*Neolecta vitellina*	DQ471037	*Neolecta vitellina*	DQ470985
	*Neolecta irregularis*	DQ842040	*Neolecta irregularis*	DQ470986
**Saccharomycetes**	*Candida glabrata*	AY218893	*Candida albicans*	DM167147
	*Saccharomyces cerevisiae*	U53879	*Saccharomyces cerevisiae*	U53879
**Taphrinomycetes**	*Protomyces inouyei*	D11377	*Protomyces inouyei*	AY548294
	*Taphrina deformans*	U00971	*Taphrina deformans*	DQ470973
**Schizosaccharomycetes**	*Schizosaccharomyces pombe*	X54866	*Schizosaccharomyces japonicus*	AATM01000140
**Chytridiomycota**	*Chytridium polysiphoniae*	AY032608	*Chytridium* sp.	DQ273831
**Zygomycota**	*Mucor racemosus*	AJ271061	*Mucor racemosus*	M26190
	*Rhizopus oryzae*	AB250174	*Rhizopus oryzae*	AACW02000152

### Sequence alignment and sequence coding

Alignments of the combined nucSSU and nucLSU rRNA sequences were performed using the Q-INS algorithm implemented in MAFFT version 6.611 [48]. The general secondary structure of *Saccharomyces cerevisiae* (U53879) was used as a reference model on the Comparative RNA Web Site (http://www.rna.icmb.utexas.edu/), and the original nucleotide data (see online Appendix 1) were recoded to produce structure sequences based on the form of base pairs [10] (see online Appendix 2).

### Phylogenetic analyses

Phylogenetic tree based on the recoding sequences was reconstructed using maximum likelihood (ML) method with the LG model (empirical substitution frequencies and gamma-distributed rates), 100 bootstrap times, via the program PhyML [49]. In addition to maximum likelihood bootstrap proportion (MLBP), phylogenetic confidence was estimated with maximum parsimony bootstrap proportions (MPBP) and Bayesian inference posterior probabilities (BIPP). MPBP value was evaluated by 1,000 replicates with 10 random addition sequences per replicate (start = stepwise addseq = random swap = tbr multrees = yes) using PAUP* 4.0b10 [50]. Bayesian inference was conducted by using MrBayes v3.0b4 with Poisson model [51]. A total of 1,000,000 initial generations were run with four Markov chains (frequency of sampling trees = 1 per 100 generations). After discarding the first 250,000 generations, the remaining trees were used to calculate BIPP based on the 50% majority-rule.

In order to evaluate the effects of recoded data on phylogenetic inference, we also performed the traditional nucleotide analyses. Under Akaike information criterion (AIC), the GTR+I+G model was estimated as the best-fit model for nucleotide data by MrModeltest [52]. The nucleotide ML analysis was done using the RAxML server with GTRCAT model (gamma distribution) and 100 bootstrap replicates [53]. BI analysis was conducted with GTR+I+G model. Subsequent phylogenetic search based on ML, BI and MP analyses was performed with the same parameter sets as those used in the analyses of structure data.

### Character state coding and analyses

Ancestral state reconstruction (ASR) is an increasingly popular method to map morphological or ecological traits onto a molecular phylogeny [54]–[56]. It assumes that there is a correlation between neutral genetic change and phenotypic change, which result in constancy of character state change rates over phylogenetic trees. Based on a single tree (e.g., a MP or ML tree) or on a Bayesian inference tree sample, ancestral state reconstruction can be performed under different optimality criteria (e.g., maximum parsimony, maximum likelihood or Bayesian approach) [56]–[58].

In this study, we traced the ascomycetous evolution with two morphological characters in addition to the molecular phylogeny, i.e. ascus dehiscence mechanism and ascoma (fruitbody) shape. The ascus dehiscence mechanism possesses six character states. They are a) *Schizosaccharomyces*-*Saccharomyces* type: with the base and top not differentiated and spore release by ascus wall deliquescence, b) *Taphrina*-*Neolecta* type: spore release by an apical slit, c) *Orbilia* type: spore release by tearing of the flattened apex of ascal wall, d) Operculate type: releasing spores by an apical lid, e) Bitunicate type: the outer wall and inner wall being differentiated during spore release, and f) Inoperculate type: releasing spores through an apical pore.

The character ascoma shape is of three states: a) Absent, b) With exposed hymenium, c) Ostiolar or sealed.

The best ML structure tree was used to trace the evolution of these characters. Considering uncertainty in tree topology and its effect on character reconstructions, the nodes with relatively weak supports (<50% MLBP) were collapsed before ASR. Reconstruction of ancestral state was done using ML (with the Markov k-state 1 parameter model) and MP criterions in Mesquite v2.0 [59].

## Supporting Information

Appendix S1 Alignments of nucleotide sequences.
(TXT)

Appendix S2 Alignments of RNA secondary structure sequences.
(TXT)
